# Practical issues in clinical scenarios involving CKD patients requiring antithrombotic therapy in light of the 2017 ESC guideline recommendations

**DOI:** 10.1186/s12916-018-1145-0

**Published:** 2018-09-19

**Authors:** Adrian Covic, Simonetta Genovesi, Patrick Rossignol, Philip A. Kalra, Alberto Ortiz, Maciej Banach, Alexandru Burlacu

**Affiliations:** 1 Nephrology Clinic, Dialysis and Renal Transplant Center – ‘C.I. Parhon’ University Hospital, Iasi, Romania; 2 ‘Grigore T. Popa’ University of Medicine, Iasi, Romania; 3 Department of Medicine and Surgery, University of Milan-Bicocca, Monza, Italy; 4 Nephrology Unit, San Gerardo Hospital, Monza, Italy; 5 Inserm, Centre d’Investigations Cliniques-Plurithématique 14-33, Inserm U1116, CHRU Nancy, Nancy, France; 6 Université de Lorraine, Association Lorraine de Traitement de l’Insuffisance Rénale (ALTIR) and F-CRIN INI-CRCT (Cardiovascular and Renal Clinical Trialists), Nancy, France; 7 The University of Manchester, Manchester Academic Health Science Centre, Salford Royal NHS Foundation Trust, Stott Lane, Salford, UK; 8 IIS-Fundacion Jimenez Diaz UAM, FRIAT and REDINREN, Madrid, Spain; 9 Department of Hypertension, Chair of Nephrology and Hypertension, Medical University of Lodz, Lodz, Poland; 10 Department of Interventional Cardiology, Cardiovascular Diseases Institute, ‘Grigore T. Popa’ University of Medicine, Iasi, Romania

**Keywords:** Anticoagulation, Antithrombotics, Bleeding, Cardiovascular disease, Chronic kidney disease, Ischemic heart disease, Risk score

## Abstract

**Background:**

The choice of the most appropriate antithrombotic regimen that balances ischemic and bleeding risks was addressed by the August 2017 European Society of Cardiologists (ESC)/European Association for Cardio-Thoracic Surgery Focused Update recommendations, which propose new evaluation scores and protocols for patients requiring a coronary stent or patients with an acute coronary syndrome, atrial fibrillation, or a high bleeding risk and indication for oral anticoagulation therapy.

**Discussion:**

Numerous questions remain regarding antithrombotic regimens and risk management algorithms for both ischemic and hemorrhagic events in patients with chronic kidney disease (CKD) in various clinical scenarios. Limitations of current studies include a general ack of advanced CKD patients in major randomized controlled trials, of evidence on algorithm implementation, and of robust assessment tools for hemorrhagic risk. Herein, we aim to analyze the ESC Update recommendations and the newly implemented risk scores (DAPT, PRECISE-DAPT, PARIS) from the point of view of CKD, providing suggestions on drug choice (which combination has the best evidence), dosage, and duration (the same or different as for non-CKD population) of antithrombotics, as well as to identify current shortcomings and to envision directions of future research.

**Conclusion:**

We provide an evidence-based perspective on the new proposed bleeding management protocol, with focus on the CKD population. Despite previous important steps on antithrombotic therapy of renal patients, there remain many unsolved questions for which our suggestions could fundament new randomized controlled trials and specific protocols.

## Background

After two decades of studying and refining dual antiplatelet therapy (DAPT), this paradigm still generates “*confusion in the community*” [1, 2] in terms of duration and its association with new drugs due to “*conflicting results and limited evidence*” [1] on specific subgroups of patients. A recent United Nations document reported that approximately 2 million patients annually require DAPT in Europe, of which almost 30% fall within different categories of chronic kidney disease (CKD) [3].

Two recent papers explored the thin line between the risks (both ischemic and hemorrhagic) and benefits (lower mortality) in the CKD setting, analyzing the existing evidence, indicating the missing information in terms of randomized controlled trials (RCTs), and highlighting the persistent need for new robust scores or algorithms to minimize hemorrhage risk while maximizing benefits [4, 5]. In August 2017, the European Society of Cardiology (ESC), in collaboration with the European Association of Cardio-Thoracic Surgery, released a focused update on DAPT [1], introducing new risk stratification tools and algorithms for the treatment of patients with percutaneous coronary interventions (PCI).

This opinion piece aims to analyze the strength and appropriateness of the new recommended risk scores (DAPT/PRECISE-DAPT) in the CKD setting as well as to critically assess the implementation of the new recommendations in patients with CKD, providing practical suggestions on drug choice (which combination is supported by the best evidence), dose (required adjustments in advanced CKD), and duration (the same/different as for non-CKD patients) of antithrombotic medication. Further, we identify current shortcomings and new directions for future research.

### Search strategy and selection criteria

Our main interest was to assess the solidity of all new recommendations from the ESC focused update document [1] in the specific subgroup of CKD patients. For each recommendation, we evaluated all the listed references from the kidney function perspective by extracting baseline estimated glomerular filtration rate (eGFR) data and the presence/absence of albuminuria of all included patients, as well as by reviewing exclusion criteria. We also performed the same evaluation as described in the ‘web addenda’ (especially the trials listed in the tables) of the ESC Update [1], assessing the existence and size of any CKD subgroup.

## Discussion

### Nephrologist perspective on new risk stratification tools for ischemia and bleeding

Approximately 28% of patients with acute coronary syndrome (ACS) have moderate CKD (eGFR 59–30 mL/min/1.73 m^2^), while 5.5% have a eGFR < 30 mL/min/1.73 m^2^ [3]. Although the percentage of patients with CKD and ACS undergoing PCI is lower than that of patients with preserved renal function [3], the number of subjects with renal failure who are candidates for DAPT is high. Since DAPT increases the risk of hemorrhagic events, it becomes mandatory to have indications on the duration of DAPT based on the patient’s bleeding risk.

The most recent ESC Update [1] proposes the use of new scores to identify the risk of intra-stent thrombosis, new myocardial infarction (MI), and major bleeding with short- (3–6 months) and long-term DAPT (≥12 months). Three new scores were recently elaborated for the stratification of thrombotic and/or hemorrhagic risk of patients with DAPT indication, namely the DAPT score [6], the PARIS score [7], and the PRECISE-DAPT score [8].

The DAPT score was created using the population of a RCT that included 468/11,648 (4.2%) CKD patients. However, the definition used to identify CKD patients was not specified. The score is a risk model for simultaneous ischemia and bleeding. Patients with a score >2 may benefit from long DAPT therapy (Table 1, calculator www.daptstudy.org). Although the presence of CKD was significantly associated with more hemorrhagic events in the study population, it was excluded as an item from the score calculator as it was not associated with thrombotic events [6].

**Table 1:** DAPT and PARIS scores (modified from [6, 7])

DAPT score		PARIS scores			
		Major bleeding		Thrombosis/MI	
Parameter	Score	Parameter	Score	Parameter	Score
Age, years		Age, years		Diabetes mellitus	
≥75	–2	<50	0	None	0
65 to <75	–1	50–59	+ 1	Non-insulin dependent	+ 1
<65	0	60–69	+ 2	Insulin dependent	+ 3
Current cigarette smoking	1	70–79	+ 3	Acute coronary syndrome	
Diabetes mellitus	1	≥80	+ 4	No	0
MI at presentation	1	Body mass index, kg/m2		Yes, Tn negative	+ 1
Prior PCI or prior MI	1	<25	+ 2	Yes, Tn positive	+ 2
Paclitaxel-eluting stent	1	25–34.9	0	Current smoking	
Stent diameter <3 mm	1	≥35	+ 2	Yes	+ 1
CHF or LVEF < 30%	2	Current smoking		No	0
Vein graft PCI	2	Yes	+ 2	eGFR < 60 mL/min	
		No	0	Present	+ 2
		Anemia		Absent	0
		Present	+ 3	Prior PCI	
		Absent	0	Yes	+ 2
		eGFR < 60 mL/min		No	0
		Present	+ 2	Prior CABG	
		Absent	0	Yes	+ 2
		Triple therapy on discharge		No	0
		Yes	+ 2		
		No	0		

*CHF* cardiac heart failure, *eGFR* estimated glomerular filtration rate, *LVEF* left ventricular ejection fraction, *MI* myocardial infarction, *PCI* percutaneous coronary intervention, *Tn* troponin

The PARIS scores were created using a registry-derived population [7]. The scores stratify PCI patients on DAPT separately for the risks of thrombosis and bleeding. Both scores include CKD (defined as eGFR < 60 mL/min/1.73 m^2^), wherein the presence of CKD increases the score by 2 points. The higher the scores, the greater the risk of intra-stent thrombosis and/or MI or the risk of bleeding (Table 1). There were 663/4190 (15.8%) and 660/8665 (7.7%) CKD patients in the discovery and validation cohorts, respectively [9]. The prevalence of CKD in the study population was significantly lower than usually reported in the literature, which is understandable since these data were obtained from registries rather than from observational/randomized trials; therefore, the relevance given by the ESC Update to the PARIS score is rather low [1] (Table 1).

**Fig. 1:**
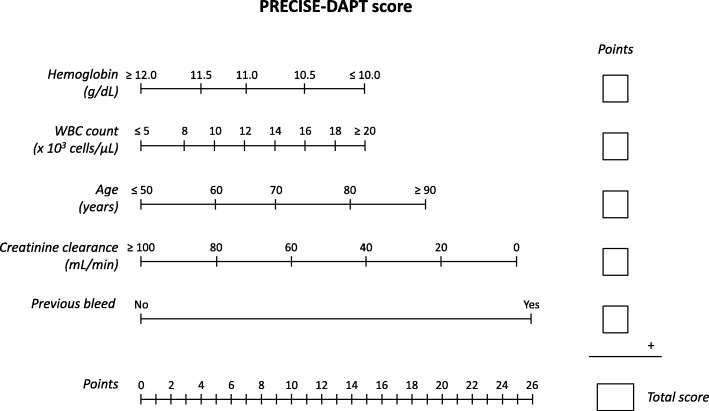
PRECISE-DAPT score (modified from [8]). *WBC* white blood cells

The PRECISE-DAPT score was created and validated in cohorts derived from RCTs [8]. The score quantifies the risk of bleeding and eGFR is included as a continuous variable. The number of CKD patients in the cohorts from which the score was created and validated was not reported, yet (as indicated in their Methods section [8]) the eGFR of the included patients was always >60 mL/min/1.73 m^2^. The other variables that compose the score are age, hemoglobin values, white blood cell count, and the presence of previous bleeding (Fig. 1 and calculator www.precisedaptscore.com). The score ranges from 0 to 100. Patients with a score >25 show an increase in bleeding events if they undergo longer DAPT without an advantage in terms of reduction of thrombotic events.

The introduction of the new scores in the ESC Update is a positive novelty for the nephrologist treating CKD patients who had an ACS. In fact, the presence of renal disease is taken into account as both a thrombotic and hemorrhagic risk factor, as a dichotomous (PARIS score) or continuous (PRECISE-DAPT score) variable. Furthermore, in the new scores, certain clinical risk factors frequently present in CKD patients (advanced age, anemia, leukocytosis, and previous bleeding) have been included.

However, the use of the new scores in CKD patients with ACS who underwent PCI presents several critical issues. The CKD population is not well defined and is poorly represented in the databases through which the scores were created and validated. The DAPT score does not include CKD as an item. The median eGFR in the cohort from which the PRECISE-DAPT was derived was 79.1 (range 60.8–98.0) mL/min/1.73 m^2^, while in the two populations in which it was validated it was 84.6 (range 67.3–102.9) mL/min/1.73 m^2^ and 87.6 (range 65.4–105.4) mL/min/1.73 m^2^, respectively [8]; therefore, the score assigned for eGFR values < 60 mL/min/1.73 m^2^ is an extrapolation. In addition, the score can be highly useful for the stratification of bleeding risk only in the presence of mild and moderate CKD. In an optimistic scenario, patients with eGFR 30 mL/min/1.73 m^2^ or 15 mL/min/1.73 m^2^, or undergoing hemodialysis would start with a moderate to high PRECISE-DAPT score (18, 22, and 25 points, respectively). In most cases, these patients are also elderly, anemic, inflamed, and with previous hemorrhagic episodes, which means that the vast majority of patients under nephrological care would show a high PRECISE-DAPT score (>25) and would therefore have to undergo a short DAPT by default. Unfortunately, this group also has a very high ischemic risk, which makes it even more difficult to decide on the duration of DAPT.

Further complications arose when solid trials proved that advanced CKD patients have an increased risk of an impaired antiplatelet effect by aspirin and clopidogrel [10, 11]. Thus, even if the above new scores indicated a longer period of DAPT, it is possible that this treatment is quite inefficient in terms of thrombotic events, especially in the advanced CKD subgroup. Fortunately, ticagrelor has a more rapid and a greater platelet inhibition than clopidogrel in G5 and G5D CKD patients [12]. More studies are needed to validate the new scores and test the new combinations of DAPT in a population prone to both a higher risk of thrombosis and more bleeding episodes.

### Discussing the brand new class I indication on proton-pump inhibitors (PPIs) and DAPT for CKD patients

Observational studies have raised concerns that several PPIs, especially omeprazole, may decrease the antiplatelet effects of clopidogrel through an inhibition of CYP2C19, resulting in an increased rate of major cardiovascular events when DAPT and PPIs were combined [13]. However, a major confounder is the fact that patients receiving PPIs frequently represent a high-risk population, having several comorbidities, including CKD, which are themselves associated with worse outcomes [13] and a higher risk of gastrointestinal bleeding [14].

The latest ESC Update granted a class I level B indication for using a PPI in combination with DAPT [1]. This recommendation mostly stems from the Clopidogrel and the Optimization of Gastrointestinal Events Trial (COGENT), which assessed the efficacy and safety of concomitant administration of clopidogrel (75 mg) and omeprazole (20 mg) in patients with coronary artery disease (CAD) (including patients with an ACS undergoing PCI), who are receiving clopidogrel plus aspirin (75 to 325 mg/d) for at least 12 months [15]. The event rate for the primary gastrointestinal end-point was reduced from 2.9% with placebo to 1.1% with omeprazole at 180 days after randomization (*P* < 0.001).

Although there was no significant difference in the rate of the primary cardiovascular end-point between the two groups (*P* = 0.98), a finding which was consistent in higher-risk subgroups, these results “*do no not rule out a clinically meaningful difference in cardiovascular events due to use of a PPI*” [15]. Importantly, as acknowledged by the authors themselves, the trial was not designed to represent high-risk patients. Since exclusion criteria comprised “*clinically significant laboratory abnormality at screening or any other condition that, in the opinion of the Investigator, precludes participation in the study*”, one may hypothesize that CKD patients were mostly excluded. Of note, neither the manuscript table of baseline patient features nor subgroup analyses reported any data related to baseline kidney function, although serum creatinine was measured at baseline according to the protocol [15]. It is therefore actually unknown whether the COGENT findings may apply to CKD patients since it is likely that no interaction with baseline CKD could be sought in this trial.

Importantly, the ESC Update acknowledges the fact that “*no randomized data comparing use vs. nonuse of PPI in patients taking aspirin and prasugrel or ticagrelor exist. However, the risk of gastro-intestinal bleeding is higher with DAPT in the form of prasugrel or ticagrelor as compared to clopidogrel*” [1]. In addition, but most importantly, one should acknowledge the fact that PPIs do not influence cerebral hemorrhages in DAPT (a significant component of major bleeding in this setting).

Li et al. [16] recently reported that PPIs significantly reduce bleeding in the context of aspirin therapy in older patients and recommended that routine co-prescription should be considered in future secondary prevention guidelines, a suggestion already implemented by the ESC Update. However, we point out that there is a close association between the use of PPIs and development of CKD, as supported by various studies [17–19]. Besides the known acute interstitial, nephritis-related kidney injury associated with PPI use [20, 21], recent research reports a non-acute kidney injury-related pathway to PPI-associated CKD [22]. Further, there is an increased risk of incident CKD, CKD progression, or end-stage renal disease (ESRD) [23] for patients on PPI medication, while observational studies report that PPI usage is associated with an increase in mortality by as much as 75% [18]. It is extremely important to note that this report included a large population of patients aged over 75 years, most probably already suffering from a declining kidney function. It is obvious that adding PPIs in this frame could potentially aggravate CKD, which would lead to various complications and costs.

Finally, we consider that, in light of the ample evidence, future guidelines should refine their class I indication regarding PPI use, at least in the advanced CKD setting. New RCTs should weigh in on both aspects and clarify whether the benefits of PPI use are greater than the worsening of renal function.

### Clinical scenario involving CKD patients with DAPT indication: which drug, for how long?

The classical paradigm of DAPT in CAD (with/without PCI) is now clearer in the general population in terms of medication, drug combinations and, most importantly, duration. There is currently a perceived divergent attitude towards a shorter (in stable CAD, low ischemic, high bleeding risk) or longer (in acute setting, high thrombotic, low bleeding risk) DAPT. Due to the numerous studies that analyzed the benefits of different intervals of DAPT and new combinations, our focus herein is to apply these new recommendations to the CKD population.

The ESC Update acknowledges the complex and debatable implication of advanced CKD on ischemic/bleeding risks, wherein a eGFR < 60 mL/min/1.73 m^2^ represents a high-risk feature of stent-driven recurrent ischemic events (see ESC Update Table five) [1], and CKD G5 and G5D pose a high hemorrhagic risk (results from PRECISE-DAPT calculator, www.precisedaptcalculator.com). The first novelty is that the PRECISE-DAPT score represents the switch sign that orientates DAPT duration toward a shorter or longer period. Even if the value of 25 represents a fragile border between a low versus high bleeding risk, this prediction model has not been prospectively tested in RCTs (especially including CKD patients). Therefore, it is probably fair to consider the decision of a low/high bleeding risk as subjective, that is, a decision that should be taken by a nephrologist-cardiologist team.

Based on the ESC Update, one can presume that, in categories G3 and G4 CKD (eGFR 15–59 mL/min/1.73 m^2^) without any other comorbidities, ischemic risk is more important than hemorrhagic risk, while in CKD G5 and G5D (eGFR < 15 mL/min/1.73 m^2^), the risk of hemorrhagic events increases, balancing the risk towards bleeding. This is the main reason for extending DAPT to longer than 12 months after an ACS with PCI if eGFR is between 30 mL/min/1.73 m^2^ and 60 mL/min/1.73 m^2^, and shortening DAPT to 6 months after ACS with PCI in patients with PRECISE-DAPT > 25 (including here CKD G5 and G5D patients).

Another novelty is that newer-generation drug-eluting stents are the preferred PCI treatment option and that there is no difference in DAPT duration for bare metal stents versus drug-eluting stents (stent type no longer matters). In addition, there is no evidence of ticagrelor and prasugrel efficacy in stable CAD (with/without PCI), but the ESC Update leaves an open door for selected cases: “*this treatment option may be considered in selected patients in whom the use of clopidogrel is unsatisfactory*” [1]. Moreover, both ticagrelor and prasugrel cannot be recommended in G5 and G5D categories of CKD [4]. Unfortunately, due to European limitations on DAPT in advanced CKD (only aspirin plus clopidogrel), practitioners face another difficult dilemma due to the frequency of poor response to clopidogrel by these patients [11, 24] and their increased risk of impaired antiplatelet effects with aspirin [10]. Despite that, small studies assessing on-treatment platelet reactivity to clopidogrel proved that the switch to standard doses of ticagrelor effectively reduced platelet activity to a level shown to be associated with fewer ischemic events [25].

In Table 2, we applied the ESC Update recommendations to a CKD population focusing on DAPT duration and drug combinations. The CKD population with CAD is divided into two main subgroups, namely the medically treated group and the PCI group; each one is then split into acute versus elective setting.

**Table 2:** Duration of treatment and drug combinations in different clinical scenarios

Clinical scenario	Status	Low bleeding risk P-D < 25		Level of evidence/References	High bleeding risk P-D > 25		Level of evidence/References
		Duration (months)	DAPT		Duration (months)	DAPT	
Medical treatment	Stable CAD	No indication for DAPT (unless overridden by prior indications)					[45]
	ACS	12–36	A + T or A + C, but not P (TRILOGY, TRITON [46, 47])	IA [48, 49] IIbB [50] for T60	1 at least, up to 6	A + C, but not A + T in medically treated ACS patients with high bleeding risk in ESC Update	IIaC^a^
PCI with stent	Stable CAD	6–30	A + C	IA IIbA [51–54]	3, lower to 1	A + C	IIaB [55, 56] IIbC [57, 58]
	ACS	12 up to indefinite	A + T or A + P or A + C	IA [46, 48, 49] IIbB [50, 59, 60] for T60^b^	6	A + C or A + T	IIaB [8, 61, 62]
BRS		Preferable not to use in persons with high bleeding risk, since DAPT duration is at least 12 months or more (A + P or A + T)					IIaC [63–65]

^a^No reference for this in ESC Update

^b^Patients >50 years old and with creatinine clearance <60 mL/min/1.73 m^2^: longer than 12 months (up to indefinite)

*A* aspirin, *ACS* acute coronary syndrome, *BRS* bioresorbable scaffolds, *C* clopidogrel, *CAD* coronary artery disease, *DAPT* dual antiplatelet therapy, *P* prasugrel, *PCI* percutaneous coronary intervention, *P-D* PRECISE-DAPT score, *T* ticagrelor, *T60* Ticagrelor 60 mg b.i.d

### Against ‘triple therapy’: low eGFR involvement in DAPT plus oral anticoagulation

There are many clinical scenarios in which patients with advanced CKD, particularly those with ESRD receiving dialysis therapy, will be prescribed oral anticoagulant therapy (OAT). Atrial fibrillation (AF) is prevalent in 8% of dialysis patients, with paroxysmal AF frequently observed [26]. Of these, approximately 1% will have non-tissue prosthetic heart valves and other patients, particularly those with renovascular disease and/or diabetes, may receive OAT for peripheral or cerebrovascular disease management; these patients are also at increased risk of developing ACS at a frequency several-fold that of age-matched non-CKD patients [27]. Modern ACS treatment protocols determine that PCI should be used in a high proportion of these patients and, in the general population, such individuals would receive DAPT for between 1 and up to 6 months [1] after PCI, with clopidogrel (but not prasugrel or ticagrelor) continued until at least 12 months (see Figure seven from the ESC Update [1]) in addition to OAT. Therefore, such patients would be exposed to a ‘triple therapy’.

Patients with advanced CKD/ESRD are at risk of major bleeding due to a number of reasons [5], with the risk being greatly increased by OAT. Before contemplating DAPT in anticoagulation patients undergoing PCI, careful consideration must be given to the impact on their bleeding risk, which will be significantly enhanced [28]. Even in the general population, the risk of major bleeding with PCI after acute MI is significantly increased with triple therapy. A Danish registry of over 40,000 patients showed an annual hospitalization rate for major bleeding of 2.6–4.3% with either aspirin, clopidogrel, or OAT therapy alone, which increased to 12% with ‘triple therapy’ [29].

The main principles of treatment in this complex situation must be to safely use antiplatelet therapy to prevent stent thrombosis whilst avoiding escalation of the bleeding risk. As the antiplatelet efficacy of aspirin is recognized to be less than that of clopidogrel, but its use in combination with clopidogrel and OAT definitely increases major bleeding risk [29, 30], patients with CKD categories G4, G5, or G5D receiving OAT and requiring PCI should receive single agent antiplatelet therapy, namely clopidogrel (see Figure seven from the ESC Update [1]).

The ESC Update states that “*in the absence of safety and efficacy data from RCTs … and worrisome bleeding signals in registries, the use of prasugrel or ticagrelor as part of triple therapy should be avoided*” [1]. On the other hand, there is still an unsolved problem regarding high clopidogrel resistance in ESRD patients [11], for which the update does not offer a solution. It is likely that future RCTs will provide innovative solutions to this complex matter.

The above data indicate that the idea of ‘triple therapy’ is not safely applicable in the advanced CKD population. Clopidogrel (or aspirin IIaA in ESC Update) should be continued for 12 months post-PCI, followed by OAT therapy alone. In patients with earlier CKD (e.g., categories G2 and G3A), although the bleeding risk is greater than in the general population, it is not as high as in categories G4 and G5 [31]; thus, the application of ‘non-CKD’ guidelines would seem appropriate, with DAPT used in combination (1–6 months) with OAT for those requiring the latter. Nevertheless, this does not consider patients with moderate CKD (category G3B). Here, the bleeding risk is increased but evidence of benefits versus risk of DAPT is also very limited. On balance, these patients should be treated like those in categories G4 and G5 (bleeding risk prevailing), with the use of only one antiplatelet agent after PCI (see Figure seven from the ESC Update [1]) plus OAT.

As has been stated in our previous work [4], evidence for the safety and benefit of novel oral anticoagulants (NOACs) in advanced CKD is very limited, with concerns regarding their metabolism in patients with minimal renal function and the associated risk of drug accumulation. Thus, where OAT is necessary, vitamin K antagonists (VKAs) are favored. A similar rationale applies to the use of clopidogrel in favor of other antiplatelet agents since evidence of their safety and efficacy in CKD categories G4 and G5 is minimal.

Even if there are no RCTs supporting NOAC use in G5D CKD patients, a 2018 meta-analysis of five observational studies showed that, among patients with advanced CKD and ESRD, the use of apixaban was associated with a lower risk of major bleeding compared to warfarin, and was found to be relatively effective with no excess risk of thromboembolic events [32]. Moreover, a 2018 KDIGO conference report “*suggests consideration of the lower dose of apixaban 2.5 mg orally twice daily in CKD G5/5D to reduce bleeding risk until clinical safety data are available*” [33]. Both of these papers showed that, in advanced CKD, apixaban is safer than warfarin in terms of bleeding and could allow the design of future studies in CKD populations requiring ‘triple therapy’ (e.g., using apixaban instead of VKAs, plus DAPT). However, with respect to ischemic events, the ESC Update stated that: “*Lower NOAC regimens as compared to those tested in approval studies are expected to decrease bleeding risk, but the trade-off between bleeding and ischaemic (i.e. stroke prevention) outcomes remains largely undefined*” [1].

In the WOEST trial [34], treatment with OAT and clopidogrel without aspirin (in patients with PCI requiring OAT) was associated with a significant reduction in bleeding complications and no increase in the rate of thrombotic events. Unfortunately, no subgroup analysis was performed on the 18% patients with a history of renal failure.

In December 2016, the PIONEER-AF-PCI trial showed that low-dose rivaroxaban plus a P2Y12 inhibitor (versus ‘triple therapy’) was associated with a lower rate of clinically significant bleeding than standard therapy, with the same efficacy on stent thrombosis prevention [35]. However, severe renal impairment (eGFR lower than 30 mL/min/1.73 m^2^) was an exclusion criterion.

The results of the 2017 RE-DUAL-PCI trial [36] demonstrated that, among AF patients who underwent PCI, the risk of bleeding was lower for those receiving dabigatran and a P2Y12 inhibitor (clopidogrel or ticagrelor) than for those receiving ‘triple therapy’ (VKA, aspirin, and P2Y12 inhibitor), without a decrease of thromboembolic events (even if eGFR < 30 mL/min/1.73 m^2^ was an exclusion criterion and an “*eGFR threshold*” for “*history of renal disease*” was not specified). We underline the fact that, while for G4 and G5 CKD categories and AF patients who underwent PCI there is no evidence that dabigatran may have an advantage over VKA, there could be a promising solution for patients with mild CKD (eGFR 30–60 mL/min/1.73 m^2^) (Table 3).

**Table 3:** Distribution of CKD patients in all four arms of RE-DUAL PCI trial [36]

Characteristic	Dabigatran 110 dual-therapy (*n* = 981), *n* (%)	Warfarin triple-therapy (*n* = 981), *n* (%)	Dabigatran 150 dual-therapy (*n* = 763), *n* (%)	Warfarin triple-therapy (*n* = 764), *n* (%)
History of renal disease	157 (16.0)	188 (19.2)	116 (15.2)	115 (15.1)

Exclusion criterion: eGFR < 30 mL/min/1.73 m^2^

Therefore, using NOACs plus a P2Y12 inhibitor in mild CKD instead of ‘triple therapy’ could be a reasonable alternative, even if these trials did not focus their analysis on the subgroup of CKD patients and despite the lack of a clear indication in the ESC Update.

### Critical analysis of new recommendations for the management of bleeding

Guidance on the management of patients who develop bleeding complications while on DAPT is supplied by the ESC Update [1], but it is not based on data from RCTs and refers to a prior Expert Consensus [37]. The key decision to be taken is whether to withhold or continue DAPT. Additionally, the type, dose, and duration of DAPT should be reassessed. These decisions should be individualized based on the relative risks of thrombosis and continuous or recurrent bleeding. A flow chart according to the severity of bleeding is provided. Guidance for the management of bleeding is especially relevant for CKD patients, mainly for those with more severe CKD. As an example, the incidence of upper gastrointestinal bleeding in hemodialysis patients was estimated at 6–33 episodes per 100 person-years, with an overall 30-day mortality of 12% [38].

Several standardized bleeding definitions from clinical trials rank the severity of bleeding in three categories (TIMI, GUSTO) or five types (BARC), one of the five being lethal bleeding (Table 4) [39–41]. The ESC Update proposes five categories, encompassing trivial, mild, moderate, severe, and life-threatening bleeding (Table 4) [1]. Mild bleeding requires medical attention, while in moderate and severe bleeding the patient is hemodynamically stable and not rapidly evolving but hemoglobin levels have fallen to >3 g/dL or >5 g/dL, respectively. Life-threatening bleeding is severe, active, and puts the patient’s life immediately at risk. Each category is associated with recommendations regarding DAPT, OAT, and general measures.

**Table 4:** Standardized bleeding definitions

From clinical trials			From guidelines	
BARC [66]	TIMI [39]	GUSTO [40]	ESC/EACTS 2017 [1]	
Type 1: Bleeding that is not actionable and does not cause the patient to seek unscheduled performance of studies, hospitalization, or treatment by healthcare professional	Minimal: Any overt bleeding event that does not meet below criteria	Mild: Bleeding that does not meet below criteria	Trivial bleeding: Any bleeding not requiring medical intervention or further evaluation	e.g., skin bruising or ecchymosis, self-resolving epistaxis, minimal conjunctival bleeding
Type 2: Any overt, actionable sign of hemorrhage that does not fit the criteria for type 3, 4, or 5, but does meet at least one of the following criteria: (1) requiring non-surgical medical intervention by a healthcare professional, (2) leading to hospitalization or increased level of care, or (3) prompting evaluation	Minor: Clinically overt bleeding resulting in Hb drop of 3 g/dL to <5 g/dL	Moderate: Bleeding requiring blood transfusion but not resulting in hemodynamic instability	Mild bleeding: Any bleeding that requires medical attention without requiring hospitalization	e.g., not self-resolving epistaxis, moderate conjunctival bleeding, genitourinary or upper/lower GI bleeding without significant blood loss, mild hemoptysis
Type 3 3a: Overt bleeding plus Hb drop of 3 g/dL to <5 g/dL or any transfusion with overt bleeding 3b: Overt bleeding plus Hb drop ≥5 g/dL, cardiac tamponade, bleeding requiring survival intervention for control or bleeding requiring intravenous vasoactive agents 3c: Intracranial hemorrhage or intraocular bleed compromising vision	Major: Fatal bleeding, intracranial bleeding or clinically overt signs of bleeding associated with a drop in Hb of ≥5 g/dL	Severe or life-threatening: Intracranial hemorrhage or bleeding resulting in substantial hemodynamic compromise requiring treatment	Moderate bleeding: Any bleeding associated with a significant blood loss (>3 g/dL Hb) and/or requiring hospitalization, which is hemodynamically stable and not rapidly evolving	e.g., genitourinary, respiratory or upper/lower GI bleeding with significant blood loss or requiring transfusion
Type 4: CABG-related bleeding, including perioperative intracranial bleeding with 48 h, reoperation after closure of sternotomy for the purpose of controlling bleeding, transfusion of ≥5 units of whole blood or packed red blood cells within a 48 h period, chest tube output ≥2 L within a 24 h period			Severe bleeding: Any bleeding requiring hospitalization, associated with a severe blood loss (>3 g/dL Hb) that is hemodynamically stable and not rapidly evolving	e.g., severe genitourinary, respiratory or upper/lower GI bleeding
Type 5: Fatal bleeding 5a: Probable fatal bleeding, no autopsy or imaging confirmation but clinically suspicious 5b: Define fatal bleeding, overt bleeding, or autopsy or imaging confirmation			Life-threatening bleeding: Any severe active bleeding putting patient’s life immediately at risk	e.g., massive overt genitourinary, respiratory or upper/lower GI bleeding, active intracranial, spinal or intraocular hemorrhage, or any bleeding causing hemodynamic instability

*BARC* Bleeding Academic Research Consortium, *CABG* coronary artery bypass graft, *EACTS* European Association for Cardio-Thoracic Surgery, *ESC* European Society of Cardiology, *GI* gastrointestinal, *GUSTO* Global Utilization of Streptokinase and Tissue plasminogen activator for Occluded coronary arteries, *Hb* hemoglobin, *TIMI* Thrombosis In Myocardial Infarction

CKD patients, especially those on hemodialysis, may have lower baseline hemoglobin values since they frequently need therapy with erythropoiesis-stimulating agents and guidelines suggest target hemoglobin levels of 9.0–10.0 g/dL to 11.5–12.0 g/dL [42]. Recent reports indicate that following the publication of KDIGO guidelines, mean hemoglobin levels have dropped, with the number of hemodialysis patients with Hb < 10 g/dL increasing from 9% in 2009 to 20% in 2012 (http://www.dopps.org/annualreport/). Thus, the potential impact of a >3 g/dL drop in hemoglobin levels (e.g., from 10 to 6 g/dL) may be higher than for individuals without baseline anemia (e.g., from 14 to 10 g/dL). Furthermore, a low hematocrit (below 30%, roughly equivalent to a hemoglobin level below 10 g/dL) favors bleeding in uremia [43]. Thus, severity thresholds based on the fall in hemoglobin levels proposed by the ESC Update to categorize the severity of bleeding may not be appropriate in CKD patients, especially in those with most advanced CKD, and decisions for action should be individualized; however, milder decreases in hemoglobin levels may be considered as thresholds to take action.

Regarding DAPT prescription upon a bleeding episode, potential actions include shortening DAPT duration, stopping DAPT, and continuing with a single antiplatelet agent, preferably with the P2Y12 inhibitor, switching to a less potent P2Y12 inhibitor (e.g., from ticagrelor or prasugrel to clopidogrel), or stopping all antithrombotic medication, at least transitorily. Since patients in CKD categories G5 and G5D are not expected to be on ticagrelor or prasugrel, the range of options for these patients is reduced.

For OAT, the range of actions includes downgrading from triple to dual therapy, preferably with clopidogrel and OAT, considering OAT discontinuation or even reversal until bleeding has stopped unless there is a very high thrombotic risk, with re-initiation when bleeding has stopped and, if the patient is in dual therapy, consider stopping antiplatelet agents. The only absolute indication to stop and reverse OAT is life-threatening bleeding, while for moderate and severe bleeding, stopping OAT may be considered until bleeding is controlled, unless the thrombotic risk is prohibitive (mechanical mitral valve, cardiac assist device) for severe bleeding or very high (mechanical heart valve, cardiac assist device, CHA2DS2-VASC score ≥ 4) for moderate bleeding. CKD patients are expected to be over-represented among those with CHA2DS2-VASC score ≥ 4, given the association of CKD with age, cardiac failure, hypertension, diabetes, stroke, and vascular disease.

Further actions may be considered depending on the severity and persistency of bleeding, including intravenous PPIs, specific hemostatic interventions depending on the site of bleeding, transfusion of platelets or red blood cells, and fluid replacement if hypotension is present. Additional options can be found in the literature for CKD G5D patients upon a severe, life-threatening bleeding episode, including the administration of desmopressin [44]; however, these are not mentioned by the ESC Update. Nevertheless, given that a potential complication of desmopressin administration is thrombosis, this should be considered a high-risk intervention. To reinitiate anticoagulation following moderate, severe, and life-threatening bleeding, guidance includes considering an International Normalization Ratio target of 2.0 − 2.5 unless there are overriding indications, such as a mechanical heart valve or cardiac assist device, as well as switching from triple to double therapy.

## Conclusions

The recent 2017 paper released by the ESC/European Association for Cardio-Thoracic Surgery to update the recommendations on modern treatment with DAPT was expected to fill the gaps of many clinical and therapeutic contexts. Among these are patients with CKD, a subgroup that raises many dilemmas of ischemic and hemorrhagic risk stratification, as well as particular therapeutic approaches. One example is the new divergent approach of DAPT’s old paradigm following PCI (short vs. long DAPT). By applying these new recommendations, algorithms, and scores (e.g., DAPT, PRECISE-DAPT, and PARIS scores) to these patients, we have identified the lack of solidity of many indications from the ESC Updated Guidelines and made suggestions based on the opinion of nephrology experts. Despite previous important steps in the antithrombotic therapy of renal patients, there remain many unsolved questions for which our suggestions could fundament new RCTs and specific protocols.

## Abbreviations

ACS: Acute coronary syndrome
AF: Atrial fibrillation
CAD: Coronary artery disease
CKD: Chronic kidney disease
DAPT: Dual antiplatelet therapy
eGFR: estimated glomerular filtration rate
ESC: European Society of Cardiology
ESRD: End-stage renal disease
MI: Myocardial infarction
NOACs: Novel oral anticoagulants
OAT: Oral anticoagulant therapy
PCI: Percutaneous coronary interventions
PPIs: Proton-pump inhibitors
RCTs: Randomized controlled trials
VKA: Vitamin K antagonists
